# Astrocytes Function as an Intermediate for Retrograde Endocannabinoid Signaling in the Suprachiasmatic Nucleus to Influence Circadian Clock Timing

**DOI:** 10.1523/ENEURO.0323-20.2020

**Published:** 2020-08-07

**Authors:** Rosalind S.E. Carney

## Abstract

**Highlighted Research Paper:** Cannabinoid Signaling Recruits Astrocytes to Modulate Presynaptic Function in the Suprachiasmatic Nucleus. Lauren M. Hablitz, Ali N. Gunesch, Olga Cravetchi, Michael Moldavan and Charles N. Allen.

Circadian rhythms regulate the timing of several homeostatic functions, such as sleep-wake cycles, hormone release, metabolism, and core body temperature. The suprachiasmatic nucleus (SCN) of the hypothalamus is the master regulator of circadian rhythms within the body. Adherence to the 24-h environmental light/dark cycle is achieved by the synchronous activity of a master pacemaker of SCN neurons in response to photic and non-photic input. This hierarchical control of synchronicity is necessary because individual SCN neurons display variation in intrinsic circadian oscillatory periods when cultured at low density (Welsh et al., 1995; Herzog et al., 1998). A cell’s circadian clock is generated by a transcriptional-translational autoregulatory loop (Ko and Takahashi, 2006; Hastings et al., 2014). In this feedback loop, master heterodimeric transcription factors CLOCK and BMAL1 regulate the expression of *Period* and *Cryptochrome* genes, which form a repressor complex that interacts with CLOCK-BMAL1 to suppress transcription of *Period* and *Cryptochrome* (King et al., 1997; Gekakis et al., 1998; Kume et al., 1999; Lee et al., 2001). The transcriptional-translational autoregulatory loop is adapted to circadian timing as PER and CRY expression increases during the day; at night, PER and CRY form a heterodimer that translocates to the nucleus to interact with the CLOCK-BMAL1 complex. Posttranslational modifications that degrade clock proteins and rhythmic chromatin remodeling also enable the circadian clock to maintain its periodicity with respect to the 24-h environmental light/dark cycle (Menet et al., 2014).

Circadian clocks are present in SCN neurons and astrocytes, as well as peripheral tissues and organs (Balsalobre et al., 1998; Abe et al., 2002; Nagoshi et al., 2004; Yoo et al., 2004; Liu et al., 2007). The expression pattern of glial fibrillary acid protein (GFAP) and the effects of inhibition of glial cell metabolic activity suggested that astrocytes may contribute to the master regulation of circadian timing within the SCN (Prosser et al., 1994; Lavialle and Servière, 1995; Santos et al., 2005). Subsequently, it was shown that SCN astrocytes have rhythms in clock gene expression and calcium signaling (Brancaccio et al., 2017) and can regulate circadian behavior (Tso et al., 2017; Brancaccio et al., 2019). The inhibitory neurotransmitter GABA is expressed by almost all of the ∼20,000 neurons within the SCN (Moore and Speh, 1993; Abrahamson and Moore, 2001). Neuronal excitation within the SCN is controlled by both glutamate release from retinohypothalamic terminals (Moore and Lenn, 1972) and presynaptic inhibition of GABA release (Acuna-Goycolea et al., 2010).

Endogenous cannabinoids (endocannabinoids) are lipid molecules, which are synthesized and released on demand from postsynaptic neurons and can affect presynaptic neurotransmitter release via retrograde signaling. Endocannabinoids exhibit diurnal variations in expression within the brain, which suggested they may contribute to circadian timing (Valenti et al., 2004). Daytime exposure to cannabinoids was known to elicit a phase advance, which is a shift forward of circadian timing (Sanford et al., 2008; Acuna-Goycolea et al., 2010). Endocannabinoids can bind to cannabinoid type 1 receptors (CB1Rs), which are also expressed by astrocytes, leading to an increase in intracellular Ca^2+^ levels within astrocytes and glutamate release (Navarrete and Araque, 2008). Within the hippocampus, this pyramidal neuron-astrocyte signaling can potentiate neurotransmitter release at CA3-CA1 synapses (Navarrete and Araque, 2010). It was also known that cortical astrocytes release ATP in an oscillatory manner dependent on circadian clock genes (Marpegan et al., 2011). However, whether astrocytes participate in retrograde signaling to regulate excitation in the SCN was unknown. In their *eNeuro* publication, Hablitz and colleagues provide support for a model in which astrocytes are identified as an intermediate in retrograde cannabinoid signaling in the SCN to influence clock timing.

The authors examined whether exposure to an artificial cannabinoid would increase intracellular Ca^2+^ levels in non-neuronal cells in the SCN. A transgenic mouse line, in which Cre recombinase expression is driven by the GFAP promoter, was used to label astrocytes. A viral construct that encodes a Cre-recombinase-activated ultrasensitive fluorescent calcium indicator (GCaMP6) was injected into the SCN of adult male and female GFAP-Cre mice. In this manner, rapid alterations in calcium levels within SCN astrocytes could be detected by calculating the change in intensity of the fluorescent signal (ΔF/F) from baseline (pretreatment) levels. Ca^2+^ levels were recorded in slices containing the SCN that were exposed to dimethylsulfoxide (DMSO; control) or the CB1/2R agonist WIN 55212-2 (WIN; dissolved in DMSO) in the recording solution. Bath application of DMSO did not affect Ca^2+^ levels in SCN astrocytes (Fig. 1*A*,*C*). Bath application of WIN resulted in an increase in fluorescence intensity, which showed that intracellular Ca^2+^ levels in astrocytes were increased following CB1R binding by WIN (Fig. 1*B*,*D*). These observations indicate that extracellular stimulation of CB1Rs using an artificial cannabinoid leads to an intracellular signaling response within astrocytes.

**Figure 1.**
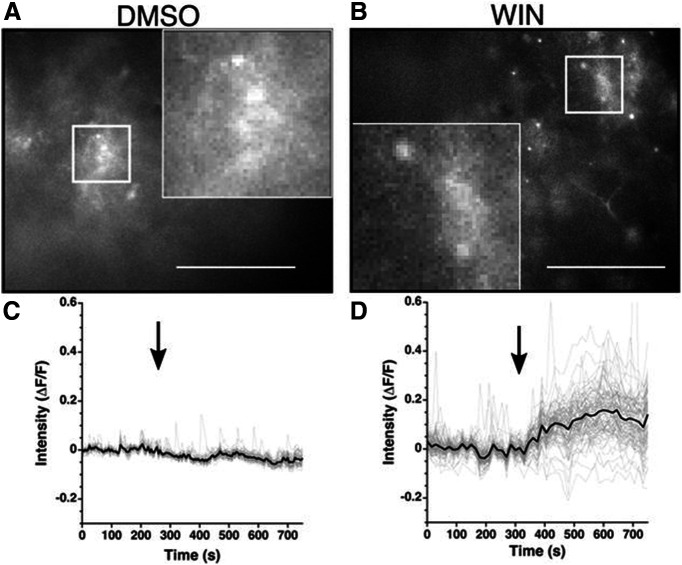
WIN activates an astrocytic Ca2^+^ signaling pathway. ***A***, ***B***, Representative GCaMP6 images from slices treated with (***A***) DMSO or (***B***) WIN. ***C***, ***D***, Representative traces showing the change in fluorescence from all regions measured (gray) and average response (black) from the slice depicted above. Black arrow indicates the beginning of treatment. Scale bar: 50 μm; images taken at 40× and the insets magnified 120×. (Adapted from Figure 3 in Hablitz *et al.*, 2020.)

**Figure 2.**
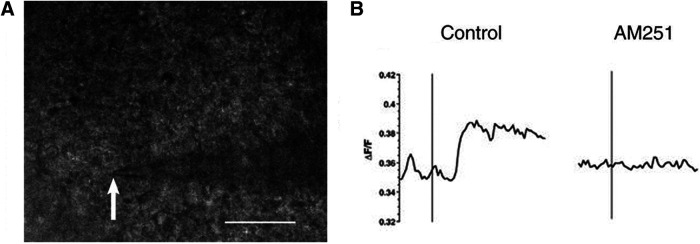
Neurons use endocannabinoid signaling to activate astrocyte Ca^2+^ signaling pathways. ***A***, Representative bright-field image from a single slice in which the white arrow indicates the placement of the microelectrode tip**. *B***, Representative intensity changes before and after depolarization of an SCN neuron (indicated by black bar) before (left, control) and during (right) AM251 treatment. White scale bar: 50 μm; images taken at 40×. (Adapted from Figure 5 in Hablitz *et al.*, 2020.)

Hablitz and colleagues next combined whole-cell patch-clamp electrophysiology of SCN neurons with GCaMP6 fluorescence recordings in local astrocytes. This approach was taken to determine whether stimulation of endocannabinoid release within a slice preparation would activate astrocyte signaling as in the prior experiment. Calcium fluorescence in astrocytes was recorded before, during, and after the depolarization of an SCN neuron (Figure 2*A*). Neuronal depolarization, which results in on-demand synthesis and release of endocannabinoids, did increase Ca^2+^ levels in astrocytes as indicated by an increase in expression levels of the GCaMP6 indicator (Figure 2*B*). The same neuronal depolarization protocol was applied following the addition of AM251 to the recording solution; AM251 is a CB1R antagonist. In the presence of AM251, GCaMP6 fluorescence intensity was similar before, during, and after SCN neuron depolarization (Figure 2*C*). These observations show that the calcium response in astrocytes is dependent on endocannabinoid signaling evoked by postsynaptic neuron stimulation.

Thus far, the results supported a model in which postsynaptic neurons release an endocannabinoid signal that binds to CB1Rs on astrocytes. Next, the authors sought to identify how activated astrocytes provide a signal to presynaptic neurons that modulates GABA release. Glutamate signaling was ruled out as the GABA receptor-mediated postsynaptic current frequency was not altered by in slices that were treated with a competitive metabotropic receptor inhibitor. Adenosine, a metabolite of ATP that is released by astrocytes, can bind to the G protein-coupled adenosine receptors. The adenosine-1 receptor (A1R)-specific antagonist 8-cyclopentyl-1,3-dipropylxanthine (DPCPX) blocked WIN-induced decreases in presynaptic mGPSC frequency. These findings suggest that the presynaptic effects of retrograde cannabinoid signaling are regulated by A1Rs on binding of adenosine, which is a metabolite of astrocytic ATP release.

A role for astrocytes as an intermediate in retrograde cannabinoid signaling was examined with respect to the known effects of cannabinoid signaling on clock timing. Organotypic slices cultures were prepared from *mPer2^Luciferase^*knock-in mice that express the PERIOD2::LUCIFERASE fusion protein (Yoo et al., 2004). After 3 d in culture, the slices were exposed to DMSO or WIN for 2 h during the day. To ensure exposure was limited to 1 h, the media were refreshed, and the cultures were maintained for an additional 3 d. Clock timing before, during, and after WIN exposure was calculated using the bioluminescence emitted from luciferase activity. Phase advances of 0.2 ± 0.1 and 2.5 ± 1.3 h occurred following exposure to DMSO or WIN, respectively (Fig. 3*A*,*B*). Using the same 1-h exposure paradigm in organotypic slice cultures, the authors showed using DPCPX that A1R inhibition did not result in a phase shift of circadian timing. When WIN was co-applied with DPCPX, WIN did not induce the phase advance that exposure to WIN alone elicited (Fig. 3*A*,*B*). Application of adenosine to the organotypic cultures using the same 1-h exposure paradigm also produced the same phase advance as observed with WIN (Fig. 3*C*,*D*). Therefore, cannabinoid signaling via adenosine-mediated activation of A1Rs is required for the phase advance to occur.

**Figure 3.**
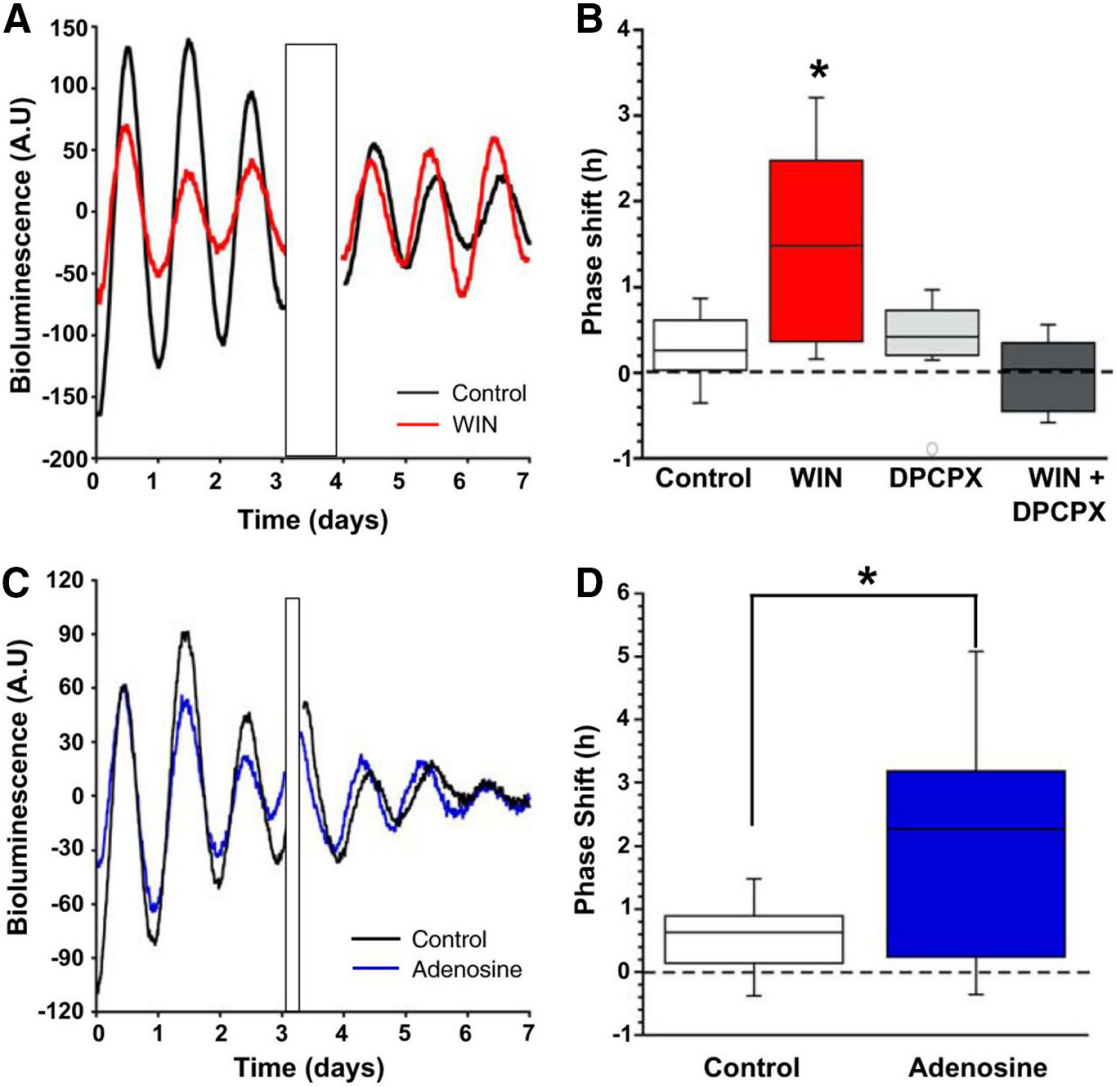
Daytime application of either WIN or adenosine phase advanced the molecular clock. ***A***, Representative bioluminescence recordings from two separate slices, one control (black), the other WIN treated (red), over 8 d. Treatment artifact is indicated by the black rectangle. ***B***, Box plot of phase shifts of all the cultures after treatment with either control (white) or WIN (red), DPCPX (light gray), or WIN+DPCPX (dark gray; *p <* *0.05). ***C***, Representative bioluminescence recordings from two separate slices, one control (black) and the other adenosine treated (blue), over 7 d. Treatment artifact is indicated by the black rectangle. ***D***, Box plot of phase shifts of SCN slice cultures after treatment with either control (black) or adenosine (blue; *p <* *0.05). (Adapted from Figure 10 in Hablitz *et al.*, 2020.)

This *eNeuro* publication is an advance in the field because it provides support to a model in which astrocytes participate in retrograde signaling by endocannabinoids to influence clock timing. Astrocytes were shown to play a crucial role in the presynaptic changes that govern a phase advance during the day. The rapid synthesis of endocannabinoids and the fact that they are not stored provides a challenge to determine which endocannabinoids are mediating this role in circadian timing. Two major endocannabinoids are anandamide and 2-arachidonoyl-glycerol (2-AG). It is known that in the hippocampus, anandamide inhibits glutamate release, whereas 2-AG mediates excitatory neurotransmission by inhibiting GABA release via CB1 receptor activation (Hájos and Freund, 2002). Hablitz and colleagues’ findings are relevant for sleep disorders for which cannabinoid therapy use is increasing (Suraev et al., 2020). Cannabinoid sleep therapies include the phytocannabinoids Δ^9^-tetrahydrocannabinol (THC) and cannabidiol (CBD), although their efficacy for sleep disorders is not yet known. It will, therefore, be important to determine whether THC and CBD are capable of producing the same astrocyte-mediated phase advances as endocannabinoids.
